# Design, Synthesis, and Evaluation of Novel Immunomodulatory Small Molecules Targeting the CD40–CD154 Costimulatory Protein-Protein Interaction

**DOI:** 10.3390/molecules23051153

**Published:** 2018-05-11

**Authors:** Damir Bojadzic, Jinshui Chen, Oscar Alcazar, Peter Buchwald

**Affiliations:** 1 Diabetes Research Institute, Miller School of Medicine, University of Miami, Miami, FL 33136, USA; d.bojadzic@med.miami.edu (D.B.); ashuichemboy@gmail.com (J.C.); o.alcazar@med.miami.edu (O.A.); 2 Molecular and Cellular Pharmacology, Miller School of Medicine, University of Miami, Miami, FL 33136, USA

**Keywords:** costimulation, drug design, immune modulation, protein-protein interaction, small molecule, TNF superfamily

## Abstract

We report the design, synthesis, and testing of novel small-molecule compounds targeting the CD40–CD154 (CD40L) costimulatory interaction for immunomodulatory purposes. This protein-protein interaction (PPI) is a TNF-superfamily (TNFSF) costimulatory interaction that is an important therapeutic target since it plays crucial roles in the activation of T cell responses, and there is resurgent interest in its modulation with several biologics in development. However, this interaction, just as all other PPIs, is difficult to target by small molecules. Following up on our previous work, we have now identified novel compounds such as DRI-C21091 or DRI-C21095 that show activity (IC_50_) in the high nanomolar to low micromolar range in the binding inhibition assay and more than thirty-fold selectivity versus other TNFSF PPIs including OX40–OX40L, BAFFR-BAFF, and TNF-R1-TNFα. Protein thermal shift (differential scanning fluorimetry) assays indicate CD154 and not CD40 as the binding partner. Activity has also been confirmed in cell assays and in a mouse model (alloantigen-induced T cell expansion in a draining lymph node). Our results expand the chemical space of identified small-molecule CD40–CD154 costimulatory inhibitors and provide lead structures that have the potential to be developed as orally bioavailable immunomodulatory therapeutics that are safer and less immunogenic than corresponding biologics.

## 1. Introduction

Cosignaling interactions, which can be either costimulatory or coinhibitory, play important roles in regulating the activation of T cells and, therefore, adequate immune responses [1]. These cell surface protein-protein interactions (PPIs) belong to two main families: the immunoglobulin superfamily (IgSF; e.g., CD28–CD80/86, CTLA4–CD80/86, or PD-L1–PD-1) and the TNFR–TNF superfamily (TNFSF; e.g., CD40–CD154, OX40–OX40L, or 4-1BB–4-1BB-L). They are particularly valuable therapeutic targets because their modulation can provide more activation- and antigen-specific effects and, hence, safer and more effective immunomodulatory agents than currently existing ones [2,3,4,5]. There are now more than 25 cosignaling pairs in both the IgSF and TNFSF, presenting a large number of possible immunomodulatory targets [6]. The high therapeutic value of these PPIs is illustrated by the fact that two recent rational drug design success stories in immunopharmacology are related to their modulation by biologics (antibodies and/or fusion proteins). Specifically, inhibition of the binding of TNF to one of its receptors resulted in five FDA-approved anti-TNF biologics (e.g., infliximab) [7], while more recently, several anticancer biologics targeting immune checkpoint (coinhibitory) PPIs, in particular PD-1–PD-L1, have received FDA approval (e.g., pembrolizumab) [8,9].

**Figure 1:**
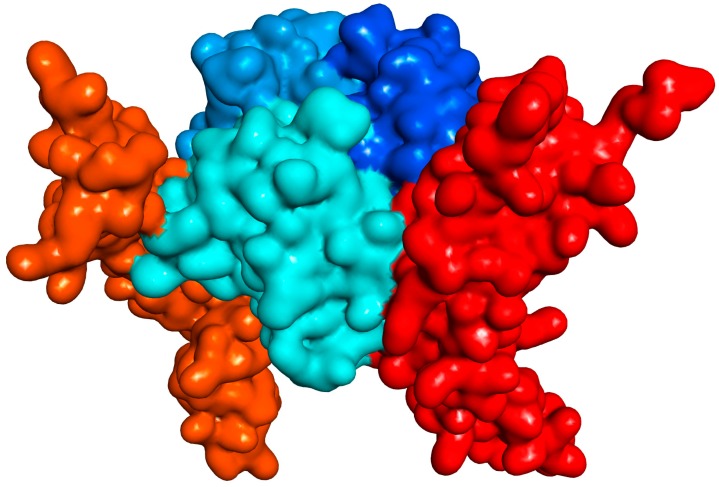
Illustration of the interacting CD40–CD154 trimer structure (human) with soft surface covered 3D protein structures (obtained using PDB ID 3QD6, which is lacking one of the CD40 monomers [11]). CD154 (CD40L) and CD40 monomers are shown in slightly different blue and red hues, respectively.

As the first TNFSF PPI identified, CD40 (TNFRSF5) and its ligand CD154 (CD40L, TNFSF5) [3] are also one of the most well studied TNFSF pairs. In the past few years, we have been focusing on the modulation of the CD40–CD154 costimulatory interaction (Figure 1) [10,11] because of its particular effectiveness in islet transplantations [12,13] as well as its promise as a target in autoimmune diseases and related inflammatory pathologies [4,14,15], including type 1 diabetes (T1D) [16,17,18]. Increasing evidence that CD40 plays a pivotal role in T1D development further makes it a promising therapeutic target for preventive interventions [16,18,19,20,21]. Studies have also indicated that CD40 serves as a possible unique biomarker for auto-aggressive T cells [16,20,21]. Accordingly, inhibition of CD40 signaling can be beneficial in chronic inflammatory diseases including autoimmune diseases, neurodegenerative disorders, graft-versus-host disease, cancer, and atherosclerosis [5]. Multiple antibodies targeting this interaction have been developed and reached various phases of preclinical or clinical development [5]. For the anti-CD154 humanized antibody ruplizumab (hu5c8), safety concerns were raised in clinical trials due to thrombotic side effects, and its further development was halted since [22,23,24]. Although platelets do express CD154, the antibody was found not to activate platelets exclusively but rather as an immunocomplex with soluble CD154, and several studies pointed to the low affinity activating Fc receptor FcγRIIa as the major cause of anti-CD154 antibody-mediated platelet activation [25,26]. Identification of the mechanism causing the thromboembolic side effects has now led to a resurgence of interest in the CD40–CD154 interaction blockade [26]. Consequently, recently developed so-called Fc-silent domain antibodies (dAbs) that lack affinity for FcγRIIa, including letolizumab and dapirolizumab pegol, were found to retain immunomodulatory activity without activating platelets [27,28].

Despite the high specificity and potency of antibodies (immunoglobulins), their development is often challenging due to species specificity and the possibility of strong immune responses elicited by their protein nature [29]. Even those that have been approved by the FDA were found to be associated with significantly more frequent post-market safety events than corresponding traditional small-molecule drugs [30]. The development of immunomodulatory biologics is further complicated by a high likelihood of unwanted adverse reactions, such as serious infections, malignancy, cytokine release syndrome, anaphylaxis, hypersensitivity, and immunogenicity [31]. For instance, one study found that 18 out of 40 licensed immunomodulatory biologics have been associated with serious infections, including reactivation of bacterial, viral, fungal, and opportunistic infections [31]. Peptides could represent a possible alternative, and some peptide inhibitors have been explored for the CD40–CD154 PPI inhibition [18,32]. However, peptides tend to be membrane impermeable as well as biologically unstable [33]; therefore, peptides, just as antibodies, are unlikely to be easily formulated for oral administration. Oral bioavailability, however, is a near prerequisite for prospective T1D preventive therapies, which have to be sufficiently patient friendly [34] in order to allow the long-term adherence and compliance needed for a successful preventive therapy [35,36]. The search for potential immunomodulatory therapies able to prevent or even revert recent-onset T1D is of particular significance as T1D remains among the few autoimmune diseases without an approved immunological therapy [37], while its incidence rate increases steadily [38,39]. All large-scale human clinical trials to date have failed to identify a therapy able to stop the functional decline of insulin-producing pancreatic β-cells, with even the most successful ones achieving no more than a few months delay in this decline [40,41,42].

We focused on identifying small-molecule compounds able to interfere with the CD40–CD154 costimulatory PPI as potential therapeutics for organ and cell transplant recipients as well as autoimmune diseases. However, small-molecule PPI inhibition is no trivial task as the interacting protein interfaces are typically featureless surfaces lacking well-defined binding pockets such as those of traditional drug targets (e.g., G-protein coupled receptors). This makes it difficult for small molecules to bind with sufficiently high energy/affinity. Nevertheless, considerable progress has been made during the last decade, and currently there are many PPI-targeting small molecules in preclinical development [43,44,45,46,47,48,49,50]. Two PPI-targeting small molecules have been recently approved by the FDA for clinical applications: venetoclax, targeting a PPI in the B cell lymphoma 2 (BCL-2) family [51,52], and lifitegrast, targeting the LFA-1–ICAM-1 interaction [48,53]. Focusing on the CD40–CD154 PPI, which is a trimer–trimer interaction as most others in TNFSF (Figure 1), we have identified the first published small-molecule inhibitors of this costimulatory PPI within the chemical space of organic dyes [54,55] and then designed a set of novel inhibitors of low micromolar potency that no longer contained color-causing chromophores [56]. Following up on this work, here, we describe the synthesis and testing of a further set of such compounds, including novel structures with improved potency, and a more detailed exploration of the corresponding structure-activity relationship.

## 2. Results

### 2.1. Design and Synthesis

**Scheme 1:**
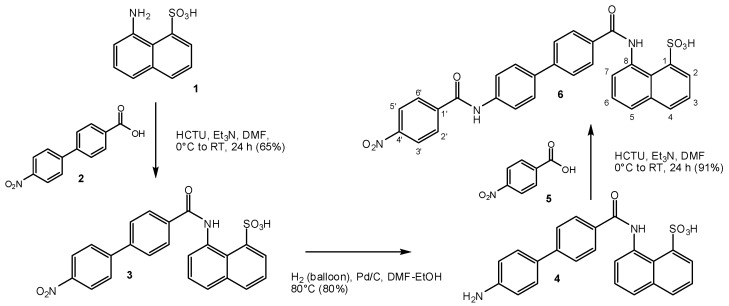
Synthesis of the compounds of the present study illustrated for compound **6**. The general coupling and reduction procedures used were similar for all compounds. For further details, see the Materials and Methods section.

**Figure 2:**
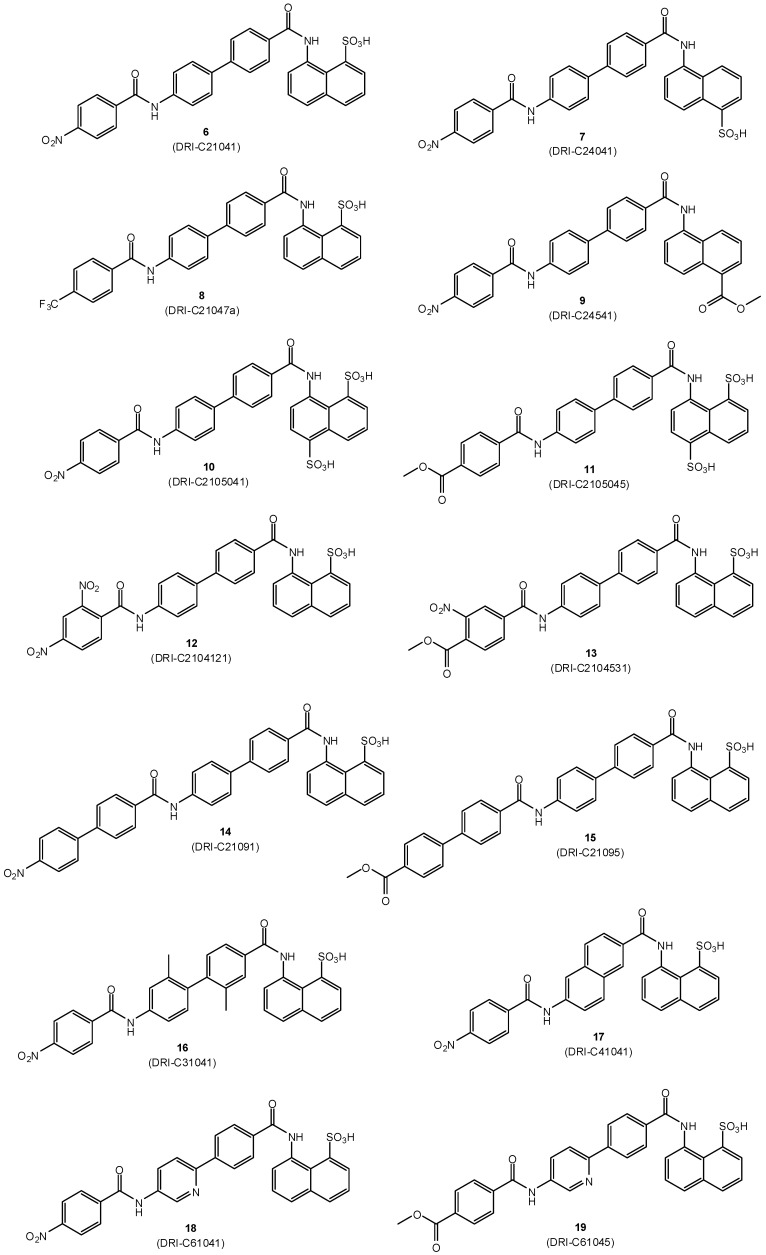
Structures of compounds **6**–**19** synthesized and investigated in the present study.

The small-molecule compounds of the present study were obtained using the same iterative design, synthesis, testing, and redesign approach as described before, and they rely on the same main structural framework that provided promising inhibitory activity for the CD40–CD154 costimulatory PPI [56]. Synthesis followed the general procedure described before [56]. Typically, it involved three steps: two amide couplings [57] and one hydrogenation [58] as illustrated in Scheme 1 for the preparation of compound **6** (DRI-C21041) starting from amine **1** and acid **2** (total overall yield 49%). Different linkers and naphthyl moieties were used as needed for other structures; all corresponding details are summarized in the Materials and Methods section. All structures synthesized and tested here (**6**–**19**) are shown in Figure 2, and corresponding details are included in Table 1.

### 2.2. Binding Inhibition Assays

**Figure 3:**
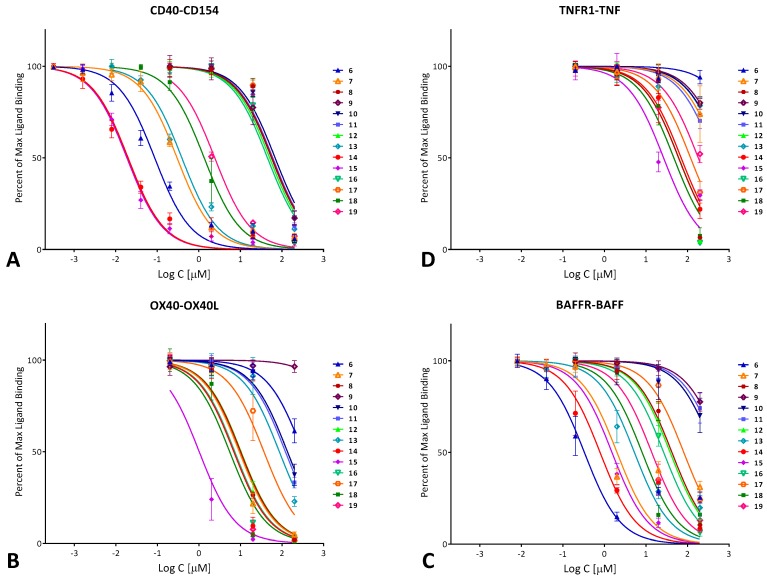
Concentration-dependent inhibition of TNF superfamily PPIs by the compounds of the present study (**6**–**19**; Table 1) in a cell-free ELISA-type assay. Data are (counterclockwise) for human CD40–CD154 (CD40L) (**A**); OX40–OX40L (**B**); BAFFR–BAFF (**C**); and TNF-R1–TNFα (**D**) and were fitted with standard binding curves. Data are average ± SD (normalized to percent binding) for *n* = 3 independent experiments with duplicates for each condition.

As a first assessment, the CD40–CD154 inhibitory activity of compounds **6**–**19** (Figure 2, Table 1) was quantified using a cell-free in vitro binding inhibition assay as described before [54,56,59]. Data fitted with typical sigmoidal concentration dependence curves (log inhibitor versus response model in GraphPad Prism, Hill slope *n* = 1) are summarized in Figure 3A. Several compounds showed activities in the mid- to high nanomolar range with **14** (DRI-C21091) and **15** (DRI-C21095) looking particularly promising with IC_50_ values of 20 and 19 nM, respectively. Polymolecular conglomeration and/or aggregation is often a cause of promiscuous inhibition in screening assays [60]. Therefore, just as in our previous work [56], we checked for this, and we confirmed for several compounds that inhibition due to aggregation is not the case by retesting inhibitory activity in the presence of a non-ionic detergent (Triton-X 100, 0.01%) as recommended for the detection of such effects [60,61]. As before, the presence of the detergent caused slight, but no significant shifts in the IC_50_s of tested compounds such as **14** (1.6×) or **15** (1.5×).

In parallel with the CD40–CD154 assay, the ability of these compounds to inhibit other TNFSFR–TNFSF PPIs, including OX40–OX40L, BAFFR–BAFF, and TNF-R1–TNF-α, was also assessed (Figure 3B–D). A few compounds showed some inhibitory activity for the OX40–OX40L or BAFFR–BAFF PPIs, but still significantly less than for CD40–CD154. Compounds of interest showed considerable selectivity toward CD40–CD154; for example, **14** and **15**, which had the highest CD154 inhibitory activity, had >30-fold selectivity against all other PPIs tested here. It is also noteworthy that **15** is the first compound we have identified so far that showed some inhibitory activity toward the TNF-R1–TNF interaction (IC_50_ of 26.5 μM; Table 1); until now, we have found no compound with activity at the <50 μM level (excluding the promiscuous PPI inhibitors [62]).

### 2.3. Binding Partner (Protein Thermal Shift)

**Figure 4:**
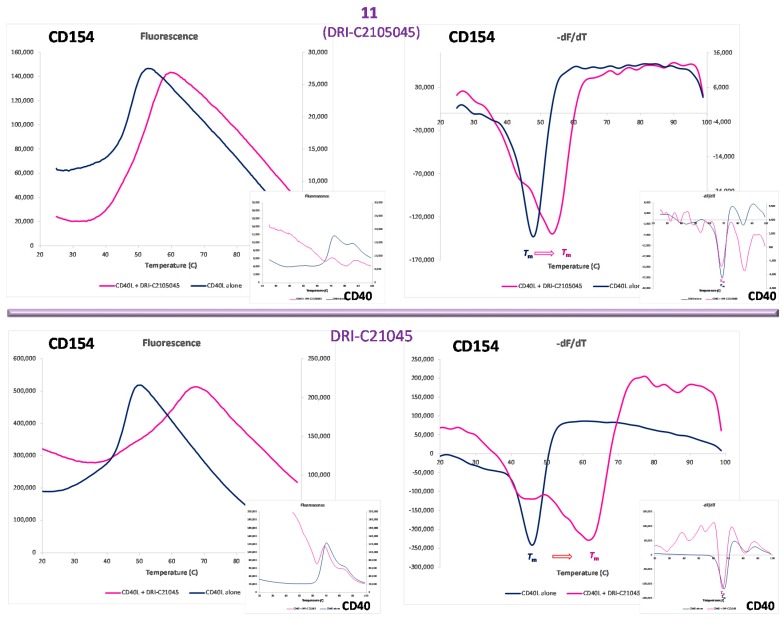
Identification of the binding partner by protein thermal shift. Differential scanning fluorimetry assay showing CD154 (CD40L) and not CD40 as the binding partner of our compounds. The presence of **11** (DRI-C2105045, 25 μM; top row) and DRI-C21045 (10 μM; bottom row) caused clear shifts in the melting temperature of the protein for CD154 (right figures; purple vs. blue line), but not for CD40 (smaller insets).

A protein thermal shift assay (differential scanning fluorimetry, also known as ThermoFluor assay) [63,64] was used to establish whether our small-molecule probes bind to CD40 or CD154 (CD40L). This technique quantifies the shift in protein stability due to ligand binding via use of a dye whose fluorescence increases when exposed to hydrophobic surfaces, which happens as the protein unfolds exposing its normally buried hydrophobic core residues. The assay allows a rapid and inexpensive evaluation of the temperature dependence of protein stability using real-time PCR instruments and only small amounts of protein, and it is sensitive enough to be used to assess small-molecule PPI interference, even as a screening assay (e.g., [65]). As shown in Figure 4, the presence of **11** as well as DRI-C21045 (a methyl ester analog of **6** from our previous work [56]) caused clear shifts in the melting temperature (*T*_m_) of the protein for CD154, but not for CD40 (purple vs. blue lines) indicating CD154 as the binding partner. As expected, the more potent compound (DRI-C21045, IC_50_ = 0.17 μM [56]) caused a larger shift than the less potent **11** (IC_50_ = 60 μM). From the present series of compounds, the disulfonic acid substituted compound **11** was used in this assay because of its significantly lesser hydrophobicity compared to the more potent compounds. Assays with the more potent, but also more hydrophobic compounds **14** or **15** were not successful because the readout of this assay relies on hydrophobicity-induced fluorescence and it was too strongly perturbed by these compounds.

### 2.4. In Vitro Activity

#### Inhibition of CD154-Induced NF-κB Activation in Sensor Cells

**Figure 5:**
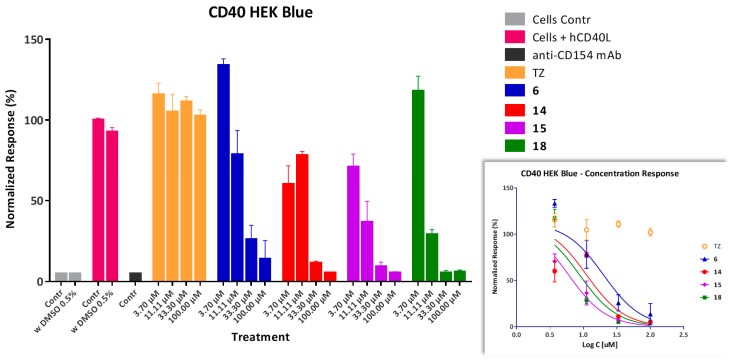
Inhibition of CD154-induced NF-κB activation in CD40 sensor cells by compounds **6**, **14**, **15**, and **18** in a concentration-dependent manner. Tartrazine (TZ) and an anti-CD154 antibody (mAb; single dose, 240 nM) were included as negative and positive controls, respectively. Data are average ± SD (normalized to CD154-activated cells alone) for *n* = 2 independent experiments with duplicates for each condition. Smaller inset at bottom right shows the concentration response on a classic semi-log scale as used to calculate the IC_50_ values.

As further confirmation of the inhibitory activity of our compounds, a cell-based assay was used with sensor cells (HEK Blue CD40) containing a secreted embryonic alkaline phosphatase (SEAP) reporter gene under the control of a promoter fused to a NF-κB binding site and transfected with CD40. In these cells, secretion of SEAP is specifically induced by the binding of CD154 to its cell-surface receptor (CD40) and the downstream activation of the corresponding NF-κB pathway. Several tested compounds such as **6**, **14**, **15**, and **18** showed concentration-dependent inhibition, as shown in Figure 5, with activities in the low micromolar range as estimated from fitting of standard sigmoid response functions (Table 1). For all tested compounds, the corresponding IC_50_ values are higher than those determined in the cell-free binding inhibition assay (Table 1) representing a loss of activity which is likely due to non-specific binding on account of the presence of proteins and cells in the assay, as well as hydrophobicity-related problems. Cytotoxicities were also evaluated in these same cells and at the same concentrations using a standard MTS assay to ensure that effects are present at non-toxic concentration levels. LC_50_ estimates obtained from fitting of standard inhibitory curves were higher than 300 μM for all compounds except **14** (55.6 μM) and **18** (131 μM) that showed some signs of toxicity and, hence, a lower safety margin (e.g., therapeutic index, TI = LC_50_/IC_50_ [66], of 5 and 15, respectively).

### 2.5. In Vivo Activity

#### Inhibition of Alloantigen-Induced Immune Response

**Figure 6:**
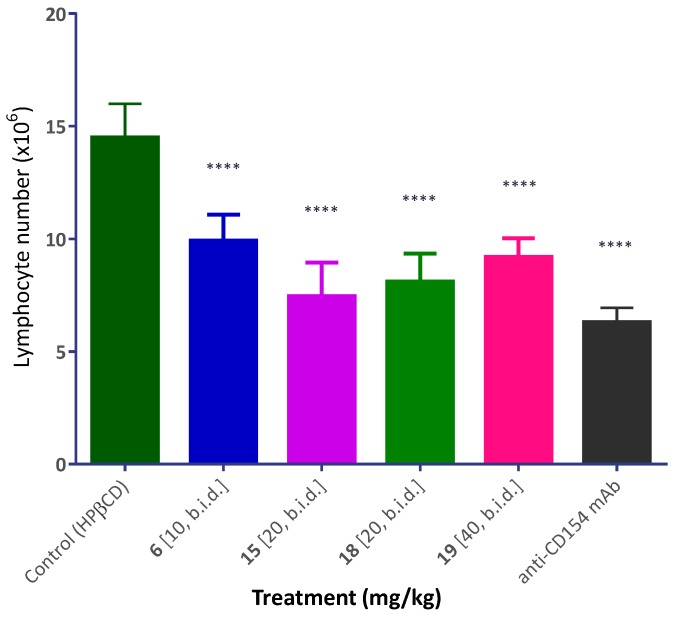
Inhibition of alloantigen-induced T cell response in a draining lymph node by compounds **6**, **15**, **18**, and **19**. Mice (Balb/c) received a footpad injection of allogeneic splenocytes (isolated from DBA-2 mice) and were treated with anti-CD154 antibody (MR-1; 20 mg/kg, day −1 and 0) or compounds as shown (10–40 mg/kg, b.i.d. from day −1 to 3, s.c. in 20% HPβCD). Draining (popliteal) lymph nodes were collected three days after the alloantigen challenge and cells were counted. Data are shown as mean ± SD. Significant differences compared to vehicle-treated controls are indicated by asterisks (**** *p* < 0.0001; one-way ANOVA followed by Dunnett’s multiple comparison test).

The most promising compounds of the present series were evaluated for in vivo activity using the same model of alloantigen-induced T cell expansion in the draining lymph nodes (DLNs) that was described before [56]. This procedure involved injection of splenocytes isolated from DBA-2 mice into the footpad of Balb/c recipients. Within three full days, this causes a significant increase in the number of cells in the draining popliteal lymph node, and this is inhibited by compounds with immunosuppressive activity. Several compounds such as **6**, **15**, and **18** (10–20 mg/kg; s.c.; b.i.d.) showed highly significant inhibitory activity (*p* < 0.0001) and at a level approaching that of the MR-1 anti-CD154 antibody used as positive control (Figure 6).

## 3. Discussion

Small molecules that can modulate costimulatory PPIs are of considerable interest as potential immunomodulatory therapeutics. They could offer useful alternatives to biologics such as antibodies especially given that small molecules are less likely to be immunogenic and have the potential of being orally bioavailable. Considerable progress along these lines has been made in the last decade even though only a few costimulatory PPI-targeting small molecules have reached preclinical (animal model) stages so far (e.g., our compounds, including the present ones, for CD40–CD154 and KR33426 for BAFFR–BAFF) and even fewer have reached clinical development (RhuDex for CD80–CD28 and CA-170 for PD-1–PD-L1) [47,50]. As a direct follow-up on our work to identify drug-like small-molecule inhibitors of the CD40–CD154 costimulatory PPI [56], here, we report a second set of molecules designed to explore the effect of different substituents and substituent positions (e.g., **7**–**9**), different linkers (e.g., **16**–**19**), longer ring chains (**14**, **15**), and increased hydrophilicity (**10**–**13**). Several new structures showed promising activity in the high nanomolar to low micromolar range in the cell-free ELISA-type assay (Figure 3A). The two most potent compounds identified so far, **14** and **15** had IC_50_ values of 20 and 19 nM in this assay (Table 1). For compound **6**, whose potency was determined by our group previously [56], we have obtained an IC_50_ of 87 nM (95% confidence interval, CI of 71–106) in the present study, slightly less than in our previous assessment (307 nM; 95% CI of 235–401) [56]. This observation indicates that the present assay may be overestimating to some extent the activity of the compounds compared to our previous work, but this much difference (approximately three-fold) could be simply due to the use of different batches of CD40 and CD154 proteins for the assay. In agreement with indications obtained for our organic dye inhibitors [54], which served as the starting point of the present design, protein thermal shift (differential scanning fluorimetry) assays here indicate that these molecules bind to CD154 (CD40L) and not CD40 (Figure 4).

For a number of compounds in the present study, there is evidence of adequate selectivity and specificity (Table 1), and several structural elements required for CD40–CD154 inhibitory activity have been identified on the basis of these data. It is clear that a chain of aromatic rings is needed for activity. Previously, we found that a two-ring linker in the middle is needed for activity [56]; here, the additional phenyl ring in compounds **14** and **15** further increased inhibitory activity. The presence of some polar substituents at both ends is also needed for activity; for example, compounds **8** with a 4′-CF_3_ substituent and **9** with a 4-COOMe substituent both lost considerable activity vs. compound **6** (Table 1). More hydrophilic structures with double substituted rings, such as the disulfonic acid **10** and **11** and the disubstituted phenyls **12** and **13**, also had diminished activities. At the 4′ position, nitro or methyl ester substituent show about similar activities (e.g., **10** vs. **11**, **14** vs. **15**, or **18** vs. **19**), but the nitro compounds tended to have increased solubility and cytotoxicity problems. In addition to the CD40–CD154 inhibitory activity, we have also assessed inhibitory activity against OX40–OX40L, BAFFR–BAFF, and TNF-R1–TNFα (Figure 3). A few molecules showed somewhat promising (i.e., low micromolar) inhibitory activity toward the BAFFR–BAFF or the OX40–OX40L (Table 1); they might serve as a starting point for a search toward more potent inhibitors of these PPIs. In fact, some off-target inhibitory activity in our compounds toward BAFFR–BAFF or OX40–OX40L could even be beneficial as additional blocking of these costimulatory interactions can further enhance immunomodulatory potential. For example, OX40 stimulation has been shown to be detrimental to allograft acceptance induced by CD40–CD154 blockade [67]; hence, its inhibition could synergize with the main effect of CD40 blockade. Nevertheless, most compounds from the chemical space investigated here show good selectivity toward CD40–CD154 inhibition indicating the potential to develop sufficiently specific inhibitors even within TNFSF PPIs. CD154, as with TNFSF ligands in general, shares relatively little sequence similarity with other TNFSF members (20–30%) [68], and this should make structure-specific targeting achievable. The CD154 homotrimer has a truncated pyramid-like shape (Figure 1) in which the interface between the trimer subunits is formed largely by hydrophobic residues, where the stacking interactions of residues such as Y170 and H224 at the center of the trimer are particularly important [11,69]. Thus, the trimerization interface of CD154 is the likely allosteric binding site of our flat, hydrophobic compounds [70], in a manner similar to BIO8898, a larger and less potent CD40–CD154 inhibitor, which has been confirmed in crystallographic studies to distort the CD154 trimer by intercalating between the monomer subunits [71].

Several compounds with promising inhibitory activity in the binding assay were tested in a cell assay (CD40-mediated activation of NF-κB sensor cells), and they all showed concentration-dependent inhibitory activity (Figure 5, Table 1). In all cases, there was loss of activity compared to the IC_50_ values observed in the cell-free binding assay–most likely due to nonspecific binding to proteins and other issues, such as limited water solubility, which are caused by the hydrophobic nature of these compounds, a frequent issue with PPI-targeting small molecules. For selected compounds, immunomodulatory activity has also been confirmed in vivo using an alloantigen-induced T cell expansion model (draining popliteal lymph node; Figure 6). Notably, all tested compounds including **6**, **15**, **18**, and **19** inhibited the alloantigen-induced T cell response in this model in statistically significant manner and at a level approaching that of the MR1 antibody, used as positive control. Compound **14** was not included in this assay as it could not be sufficiently solubilized in the vehicle to permit comparable in vivo dosing. It is also noteworthy that while the present compounds are not very small (molecular weights in the 550 to 650 Da range), they are still relatively small structures compared to typical PPI inhibitors, which often need to have larger structures to achieve sufficient activity and are often in flagrant violation of the widely used “rule-of-five” criteria, which requires (among others) MW < 500 [72]. In the last two decades, these criteria have been commonly used as a guide to ensure oral bioavailability and an adequate pharmacokinetic profile. Nevertheless, an increasing number of new drugs have been launched recently (including the two small-molecule PPI inhibitors discussed earlier) that significantly violate these empirical rules proving that oral bioavailability can be achieved even in the “beyond rule-of-five” chemical space [73]. Accordingly, our results provide further proof for the feasibility of small-molecule inhibition of the CD40–CD154 PPI and provide a first map of the structure-activity relationships of the corresponding chemical space of drug-like structures.

## 4. Materials and Methods

### 4.1. Materials

All chemicals and reagents used were obtained from Sigma-Aldrich (St. Louis, MO, USA) except as indicated. Recombinant receptors (CD40:Fc, OX40:Fc, BAFFR:Fc, and TNF-R1:Fc) and FLAG-tagged ligands (CD154, OX40L, BAFF, and TNF-α) were obtained from Enzo Life Sciences (San Diego, CA, USA). CD40L used in the thermal shift assay was from Peprotech (Rocky Hill, NJ, USA). The monoclonal anti-human CD154 (clone 40804) was obtained from R&D Systems (Minneapolis, MN, USA).

### 4.2. Chemistry

Commercial grade reagents and solvents were purchased from VWR (Radnor, PA, USA) and Sigma-Aldrich (St. Louis, MO, USA) and directly used without further purification. All reactions were carried out in oven- or flame-dried glassware under an atmosphere of dry argon unless otherwise noted. Except as otherwise indicated, all reactions were magnetically stirred and monitored by analytical thin-layer chromatography (TLC) using Merck (Kenilworth, NJ, USA) pre-coated silica gel plates with F_254_ indicator. Visualization was accomplished by UV light (256 nm) with a combination of potassium permanganate and/or vanillin solution as an indicator. Flash column chromatography was performed according to the method of Still [74] using silica gel 60 (mesh 230–400; EMD Milipore, Billerica, MA, USA).

General synthetic procedures were the same as described before [56]; details are briefly summarized below for all individual compounds and intermediaries. Newly synthesized compounds were characterized with ^1^H-NMR, ^13^C-NMR, high-resolution mass spectrometry (HRMS), and infrared (IR) spectroscopy–detailed data are provided below. Chemical shifts are reported in ppm relative to TMS. DMSO-*d*_6_ (2.50 ppm) was used as a solvent for ^1^H-NMR and ^13^C-NMR. ^1^H-NMR and ^13^C-NMR spectra were recorded on Bruker Avance 300 (300 MHz ^1^H), 400 (400 MHz ^1^H, 100 MHz ^13^C), and 500 (500 MHz ^1^H, 125 MHz ^13^C). Chemical shift values (*δ*) are reported in ppm relative to Me_4_Si (*δ* 0.0 ppm) unless otherwise noted. Proton spectra are reported as *δ* (multiplicity, coupling constant *J*, number of protons). Multiplicities are indicated by *s* (singlet), *d* (doublet), *t* (triplet), *q* (quartet), *p* (quintet), *h* (septet), *m* (multiplet), and *br* (broad). IR spectra were recorded with a FT-IR spectrophotometer Paragon 1000 (PerkinElmer). Mass spectra were obtained at the Mass Spectrometry Laboratory, Department of Chemistry, University of Florida (Gainesville, FL, USA). Low-resolution ES (electron spray) mass spectra were carried out with Finnigan LCQ DECA/Agilent 1100 LC/MS mass spectrometer (Thermo Fisher Scientific, Waltham, MA, USA). High-resolution mass spectra were recorded on an Agilent 6220 ESI TOF (Santa Clara, CA, USA) mass spectrometer. Analysis of sample purity was performed on an Agilent (Palo Alto, CA, USA) 1100 series HPLC system with a ThermoScientific Hypurity C8 (5 μm; 2.1 × 100 mm + guard column). HPLC conditions were as follows: solvent A = water with 2 mM ammonium acetate, solvent B = methanol with 2 mM ammonium acetate, and flow rate = 0.2 mL/min. Compounds were eluted with a gradient of A/B = 80:20 at 0 min to 0:100 at 50 min. Purity was determined via integration of UV spectra at 254 nm, and all tested compounds have a purity of ≥95%. All target compounds **6**−**19** were tested as triethylamine salts unless otherwise stated.

*8-(4′-Nitrobiphenyl-4-ylcarboxamido)naphthalene-1-sulfonic acid* (**3**) *and the general procedure for coupling.* 4′-Nitro[1,1′-biphenyl]-4-carboxylic acid (**2**) was synthesized by two steps as described in the literature [75]. For the synthesis of **3** and as a general procedure of coupling a modified version of the coupling reaction from reference [57] was used (Scheme 1). Under an argon atmosphere, trimethylamine (1.74 mL, 12.5 mmol) was added dropwise to a mixture of 4′-nitro[1,1′-biphenyl]-4-carboxylic acid (**2**) (1.63 g, 6.7 mmol), *O*-(6-chlorobenzotriazol-1-yl)-*N*,*N*,*N*′,*N*′-tetramethyluronium hexafluorophosphate HCTU (2.7 g, 6.5 mmol) and DMF (10 mL) at 0 °C and the resulting reaction mixture was stirred for 1 h at the same temperature. Subsequently, 8-amino-1-naphthalenesulfonic acid **1** (1.50 g, 6.7 mmol) was added at the same temperature. The resulting reaction mixture was allowed to stir overnight at room temperature (RT). Diethyl ether (50 mL) was added to the reaction mixture, and a yellow precipitate formed. This precipitate was collected by filtration and washed with diethyl ether (3 × 10 mL) to afford the triethylamine salt of **3** as a yellow solid (2.4 g, 66%). ^1^H-NMR (500 MHz, DMSO-*d*_6_): *δ* 12.70 (s, 1H), 8.79 (br, 1H), 8.38–8.29 (m, 5H), 8.20 (d, *J* = 7.6 Hz, 1H), 8.09 (d, *J* = 8.8 Hz, 2H), 8.02 (d, *J* = 8.1 Hz, 1H), 7.95 (d, *J* = 8.4 Hz, 2H), 7.83 (d, *J* = 7.8 Hz, 1H), 7.59 (t, *J* = 7.9 Hz, 1H), 7.48 (t, *J* = 7.6 Hz, 1H), 3.08 (q, *J* = 7.2 Hz, 6H), 1.15 (t, *J* = 7.3 Hz, 9H); ^13^C-NMR (125 MHz, DMSO-*d*_6_): δ 164.8, 147.0, 145.8, 141.8, 140.1, 135.9, 135.8, 133.2, 131.9, 128.9, 128.1, 127.4, 127.0, 126.1, 125.3, 124.2, 124.1, 124.0, 123.0, 45.8, 8.6; FTIR (neat) ν_max_ 3028, 2735, 1668, 1596, 1515, 1494, 1480, 1429, 1393, 1339, 1328, 1280, 1231, 1186, 1162, 1126, 1110, 1039, 1010, 924, 894, 869, 854, 843, 826, 788, 763, 740, 692 cm^−1^; HRMS (ESI) [M + H]^+^ calcd. for C_23_H_17_N_2_O_6_S^+^, 449.0802; found, 449.0781.

*8-(4′-Aminobiphenyl-4-ylcarboxamido)naphthalene-1-sulfonic acid* (**4**) *and the general procedure for hydrogenation*. For the synthesis of **4** and as a general procedure of coupling a modified version of the hydrogenation reaction from reference [58] was used (Scheme 1). A mixture of 8-(4′-nitrobiphenyl-4-ylcarboxamido)naphthalene-1-sulfonic acid (**3**) (2.8 g, 5.1 mmol) and 10% Pd on carbon (27 mg) in a solvent mixture of EtOH (2.0 mL) and DMF (1.0 mL) was hydrogenated (H_2_ balloon) at 80 °C for 3.5 h. The reaction mixture was filtered via a short pad of Celite^®^, concentrated *in vacuo*, and recrystallized from MeOH to afford the triethylamine salt of **4** as a white solid (2.3 g, 86%). ^1^H-NMR (500 MHz, DMSO-*d*_6_): *δ* 12.56 (s, 1H), 8.80 (br, 1H), 8.31 (dd, *J* = 1.1, 6.1 Hz, 1H) , 8.25–8.12 (m, 3H), 8.00 (d, *J* = 7.2 Hz, 1H), 7.80 (d, *J* = 7.3 Hz, 1H), 7.67 (d, *J* = 8.3 Hz, 2H), 7.57 (t, *J* = 7.8 Hz, 1H), 7.49 (d, *J* = 7.0 Hz, 2H), 7.47 (t, *J* = 7.7 Hz, 1H), 6.68 (d, *J* = 8.4 Hz, 2H), 5.33 (s, 2H), 3.05 (q, *J* = 7.2 Hz, 6H), 1.14 (t, *J* = 7.3 Hz, 9H); ^13^C-NMR (125 MHz, DMSO-*d*_6_): δ 165.2, 148.9, 143.1, 141.8, 135.7, 133.4, 132.5, 131.8, 128.6, 127.4, 127.3, 126.3, 125.7, 125.3, 124.6, 124.1, 123.9, 123.0, 114.2, 45.7, 8.6; FTIR (neat) ν_max_ 3431, 3338, 3227, 3006, 2712, 1648, 1602, 1531, 1492, 1474, 1429, 1397, 1331, 1284, 1227, 1196, 1184, 1161, 1061, 1036, 1008, 921, 891, 823, 788, 761, 725, 704, 663 cm^−1^; HRMS (ESI) [M + H]^+^ calcd. for C_23_H_19_N_2_O_4_S^+^, 419.1060; found, 419.1058.

*8-(4′-(4-Nitrobenzamido)biphenyl-4-ylcarboxamido)naphthalene-1-sulfonic acid* (**6**). Preparation of compound **6** followed the general synthetic scheme of Scheme 1 via intermediaries **3** and **4**. The general procedure for coupling as described for **3** was followed with 4-nitrobenzoic acid (**5**) (0.50 g, 3.0 mmol) and 8-(4′-aminobiphenyl-4-ylcarboxamido)naphthalene-1-sulfonic acid (**4**) (1.20 g, 2.3 mmol) to give the triethylamine salt of the title compound **6** as a yellow solid (1.35 g, 87%) (99.9% pure by HPLC analysis (UV spectra at 254 nm)). ^1^H-NMR (500 MHz, DMSO-*d*_6_): *δ* 12.64 (s, 1H), 10.75 (s, 1H), 8.85 (br, 1H), 8.39 (d, *J* = 8.1 Hz, 2H), 8.36–8.16 (m, 6H), 8.02 (d, *J* = 8.2 Hz, 1H), 7.95 (d, *J* = 8.2 Hz, 2H), 7.91–7.81 (m, 5H), 7.59 (t, *J* = 7.7 Hz, 1H), 7.49 (t, *J* = 7.5 Hz, 1H), 3.07 (q, *J* = 7.2 Hz, 6H), 1.15 (t, *J* = 7.1 Hz, 9H); ^13^C-NMR (125 MHz, DMSO-*d*_6_): δ 165.1, 164.1, 149.2, 142.0, 141.8, 140.6, 138.8, 135.8, 134.9, 134.2, 133.3, 132.0, 129.4, 128.8, 127.5, 127.3, 126.0, 125.9, 125.4, 124.4, 124.1, 123.7, 123.0, 120.8, 45.7, 8.7; FTIR (neat) ν_max_ 3360, 3017, 2714, 1679, 1666, 1592, 1521, 1489, 1432, 1416, 1398, 1340, 1321, 1279, 1235, 1194, 1152, 1131, 1102, 1038, 1009, 929, 895, 864, 852, 824, 761, 708, 675, 661 cm^−1^; HRMS (ESI) [M − H]^−^ calcd. for C_30_H_20_N_3_O_7_S^−^, 566.1027; found, 566.1054.

*5-(4′-(4-Nitrobenzamido)-[1,1′-biphenyl]-4-carboxamido)naphthalene-1-sulfonic acid* (**7**)*.* The general procedure for the coupling reaction as described earlier was followed with 4′-(4-nitrobenzamido)-[1,1′-biphenyl]-4-carboxylic acid (181 mg, 0.50 mmol) and 5-aminonaphthalene-1-sulfonic acid (112 mg, 0.50 mmol) to give the triethylamine salt of the title compound as a yellow solid (210 mg, 63%) (>99.9% pure by HPLC analysis (UV spectra at 254 nm)). ^1^H-NMR (500 MHz, DMSO-*d*_6_): *δ* 10.73 (s, 1H), 10.49 (s, 1H), 8.86 (br, 1H), 8.39 (d, *J* = 8.4 Hz, 2H), 8.30–8.15 (m, 5H), 8.05–7.88 (m, 6H), 7.84 (d, *J* = 8.4 Hz, 2H), 7.77 (d, *J* = 8.8 Hz, 1H), 7.65 (d, *J* = 7.1 Hz, 1H), 7.58 (t, *J* = 7.9 Hz, 1H), 3.08 (q, *J* = 6.8 Hz, 6H), 1.16 (t, *J* = 7.2 Hz, 9H); ^13^C-NMR (125 MHz, DMSO-*d*_6_): δ 165.8, 164.0, 149.2, 144.3, 142.5, 140.5, 138.8, 134.6, 133.7, 132.9, 130.0, 129.8, 129.3, 128.5, 127.2, 126.3, 126.2, 125.0, 124.8, 124.53, 124.46, 123.9, 123.6, 120.8, 45.8, 8.6; HRMS (ESI) [M − H]^−^ calcd. for C_30_H_20_N_3_O_7_S^−^, 566.1027; found, 566.1025.

*8-(4′-(4-(trifluoromethyl)benzamido)biphenyl-4-ylcarboxamido)naphthalene-1-sulfonic acid* (**8**)*.* The general procedure for the coupling reaction as described earlier was followed with 4-(trifluoromethyl)benzoic acid (114 mg, 0.60 mmol) and 8-(4′-amino-[1,1′-biphenyl]-4-carboxamido)naphthalene-1-sulfonic acid **4** (260 mg, 0.50 mmol) to give the triethylamine salt of the title compound as a white solid (218 mg, 63%) (96.1% pure by HPLC analysis (UV spectra at 254 nm)). ^1^H-NMR (500 MHz, DMSO-*d*_6_): *δ* 12.65 (s, 1H), 10.61 (s, 1H), 8.79 (br, 1H), 8.32 (d, *J* = 7.2 Hz, 1H), 8.27 (d, *J* = 8.1 Hz, 2H), 8.24–8.16 (m, 3H), 8.02 (d, *J* = 8.1 Hz, 1H), 7.95 (d, *J* = 8.6 Hz, 2H), 7.93 (d, *J* = 8.2 Hz, 2H), 7.88–7.78 (m, 5H), 7.59 (t, *J* = 7.8 Hz, 1H), 7.48 (t, *J* = 7.5 Hz, 1H), 3.06 (q, *J* = 7.3 Hz, 6H), 1.14 (t, *J* = 7.3 Hz, 9H); ^13^C-NMR (125 MHz, DMSO-*d*_6_): δ 165.1, 164.5, 142.0, 141.8, 138.8, 138.7, 135.8, 134.8, 134.1, 133.3, 131.8, 131.8, 131.4 (q, *J*_C-F_ = 31.8 Hz, 1C), 128.8, 128.6, 127.4, 127.1, 125.9, 125.8, 125.4, 125.4, 125.4, 125.4, 125.3, 125.0, 124.2, 124.0, 123.9 (q, *J*_C-F_ = 265.8 Hz, 1C), 123.0, 122.8, 120.7, 45.8, 8.6; HRMS (ESI) [M − H]^−^ calcd. for C_31_H_20_F_3_N_2_O_5_S^−^, 589.1051; found, 589.1045.

*Methyl 5-(4′-(4-nitrobenzamido)-[1,1′-biphenyl]-4-carboxamido)-1-naphthoate* (**9**). The general procedure for the coupling reaction as described earlier was followed with 4′-(4-nitrobenzamido)-[1,1′-biphenyl]-4-carboxylic acid (181 mg, 0.50 mmol) and methyl 5-amino-1-naphthoate (101 mg, 0.50 mmol) to give the title compound as a yellow solid (150 mg, 55%) (>99.9% pure by HPLC analysis (UV spectra at 254 nm)). ^1^H-NMR (500 MHz, DMSO-*d*_6_): *δ* 10.72 (s, 1H), 10.58 (s, 1H), 8.67 (d, *J* = 6.8 Hz, 1H), 8.40 (d, *J* = 6.3 Hz, 2H), 8.35–8.13 (m, 6H), 8.05–7.80 (m, 6H), 7.77–7.59 (m, 3H), 3.97 (s, 3H); ^13^C-NMR (125 MHz, DMSO-*d*_6_): δ 167.4, 165.9, 164.0, 149.2, 142.7, 140.5, 138.9, 134.6, 134.5, 132.7, 131.1, 129.8, 129.8, 129.3, 128.6, 127.4, 127.3, 127.2, 126.2, 124.9, 124.6, 123.6, 120.8, 52.4; HRMS (ESI) [M + Na]^+^ calcd. for C_32_H_23_N_3_NaO_6_^+^, 568.1479; found, 568.1471.

*4-(4′-(4-Nitrobenzamido)-[1,1′-biphenyl]-4-carboxamido)naphthalene-1,5-disulfonic acid* (**10**). The general procedure for the coupling reaction as described earlier was followed with 4-nitrobenzoyl chloride (102 mg, 0.55 mmol) and 4-(4′-amino-[1,1′-biphenyl]-4-carboxamido)naphthalene-1,5-disulfonic acid (249 mg, 0.50 mmol) to give the title compound as a yellow solid (270 mg, 83%). ^1^H-NMR (500 MHz, DMSO-*d*_6_): *δ* 12.71 (s, 1H), 10.78 (s, 1H), 9.13 (d, *J* = 8.7 Hz, 1H), 8.39 (d, *J* = 8.4 Hz, 2H), 8.33 (d, *J* = 7.2 Hz, 1H), 8.29–8.20 (m, 4H), 8.09 (d, *J* = 8.0 Hz, 1H), 8.06 (d, *J* = 8.2 Hz, 1H), 7.97 (d, *J* = 8.3 Hz, 2H), 7.84 (d, *J* = 8.2 Hz, 4H), 7.48 (t, *J* = 7.6 Hz, 1H); ^13^C-NMR (125 MHz, DMSO-*d*_6_): δ 165.2, 164.0, 149.2, 142.0, 141.8, 141.0, 140.6, 138.8, 134.9, 134.5, 134.2, 131.6, 130.8, 129.4, 128.8, 127.2, 125.9, 124.6, 123.6, 123.5, 122.4, 120.9; HRMS (ESI) [M − H]^−^ calcd. for C_30_H_20_N_3_O_10_S_2_^−^, 646.0596; found, 646.0602.

*4-(4′-(4-(Methoxycarbonyl)benzamido)-[1,1′-biphenyl]-4-carboxamido)naphthalene-1,5-disulfonic acid* (**11**). The general procedure for the coupling reaction as described earlier was followed with methyl 4-(chlorocarbonyl)benzoate (119 mg, 0.60 mmol) and 4-(4′-amino-[1,1′-biphenyl]-4-carboxamido)naphthalene-1,5-disulfonic acid (249 mg, 0.50 mmol) to give the title compound as a white solid (258 mg, 78%). ^1^H-NMR (500 MHz, DMSO-*d*_6_): *δ* 12.71 (s, 1H), 10.59 (s, 1H), 9.14 (d, *J* = 8.6 Hz, 1H), 8.34 *J* = 7.2 Hz, (d, 1H), 8.25 (d, *J* = 8.0 Hz, 2H), 8.16–8.04 (m, 6H), 7.95 (d, *J* = 8.4 Hz, 2H), 7.88–7.79 (m, 4H), 7.49 (t, *J* = 7.5 Hz, 1H), 3.91 (s, 3H); ^13^C-NMR (125 MHz, DMSO-*d*_6_): δ 165.7, 165.2, 164.8, 142.0, 141.8, 141.1, 139.0, 138.9, 134.7, 134.4, 134.1, 132.1, 131.6, 130.8, 129.2, 128.8, 128.2, 127.1, 125.8, 124.6, 123.5, 122.3, 120.8, 52.5; HRMS (ESI) [M − H]^−^ calcd. for C_32_H_23_N_2_O_10_S_2_^−^, 659.0800; found, 659.0818.

*8-(4′-(2,4-Dinitrobenzamido)-[1,1′-biphenyl]-4-carboxamido)naphthalene-1-sulfonic acid* (**12**). The general procedure for the coupling reaction as described earlier was followed with 2,4-dinitrobenzoic acid (138 mg, 0.65 mmol) and 8-(4′-amino-[1,1′-biphenyl]-4-carboxamido)naphthalene-1-sulfonic acid **4** (260 mg, 0.50 mmol) to give the triethylamine salt of the title compound as a yellow solid (219 mg, 61%) (>99.9% pure by HPLC analysis (UV spectra at 254 nm)). ^1^H-NMR (500 MHz, DMSO-*d*_6_): *δ* 12.62 (s, 1H), 11.03 (s, 1H), 8.84 (d, *J* = 1.9 Hz, 1H), 8.65 (dd, *J* = 8.3, 2.1 Hz, 1H), 8.31 (d, *J* = 7.2 Hz, 1H), 8.27 (d, *J* = 8.0 Hz, 2H), 8.21 (d, *J* = 7.6 Hz, 1H), 8.12 (d, *J* = 8.3 Hz, 1H), 8.01 (d, *J* = 8.3 Hz, 1H), 7.88–7.77 (m, 7H), 7.59 (t, *J* = 7.8 Hz, 1H), 7.48 (t, *J* = 7.5 Hz, 1H); ^13^C-NMR (125 MHz, DMSO-*d*_6_): δ 165.1, 162.5, 148.0, 146.6, 141.9, 141.7, 138.3, 137.2, 135.8, 135.2, 134.2, 133.3, 131.9, 131.1, 128.8, 128.5, 127.4, 125.9, 125.9, 125.3, 124.2, 124.0, 123.0, 120.2, 119.8; HRMS (ESI) [M + Na]^+^ calcd. for C_30_H_20_N_4_NaO_9_S^+^, 635.0843; found, 635.0860.

*8-(4′-(4-(Methoxycarbonyl)-3-nitrobenzamido)biphenyl-4-ylcarboxamido)naphthalene-1-sulfonic acid* (**13**). The general procedure for the coupling reaction as described earlier was followed with 4-(methoxycarbonyl)-3-nitrobenzoic acid (146 mg, 0.65 mmol) and 8-(4′-amino-[1,1′-biphenyl]-4-carboxamido)naphthalene-1-sulfonic acid **4** (260 mg, 0.50 mmol) to give the triethylamine salt of the title compound as a yellow solid (260 mg, 72%) (>99.9% pure by HPLC analysis (UV spectra at 254 nm)). ^13^C-NMR (125 MHz, DMSO-*d*_6_): δ 165.0, 164.7, 162.6, 147.6, 141.9, 141.8, 138.5, 135.8, 135.1, 134.2, 133.3, 132.7, 131.8, 130.2, 128.8, 128.6, 127.4, 127.2, 125.9, 125.9, 125.3, 124.2, 124.0, 123.4, 123.0, 120.8, 53.4, 45.8, 8.6; HRMS (ESI) [M − H]^−^ calcd. for C_32_H_22_N_3_O_9_S^−^, 624.1082; found, 624.1066.

*8-(4′-(4′-Nitro-[1,1′-biphenyl]-4-carboxamido)-[1,1′-biphenyl]-4-carboxamido)naphthalene-1-sulfonic acid* (**14**). The general procedure for the coupling reaction as described earlier was followed with 4′-nitro-[1,1′-biphenyl]-4-carboxylic acid **2** (0.73 g, 3.0 mmol) and 8-(4′-amino-[1,1′-biphenyl]-4-carboxamido)naphthalene-1-sulfonic acid **4** (1.30 g, 2.5 mmol) to give the triethylamine salt of the title compound as a yellow solid (1.67 g, 90%) (>98.7% pure by HPLC analysis (UV spectra at 254 nm)). ^1^H-NMR (500 MHz, DMSO-*d*_6_): *δ* 12.65 (s, 1H), 10.50 (s, 1H), 8.81 (br, 1H), 8.39–8.30 (m, 3H), 8.28 (d, *J* = 8.1 Hz, 2H), 8.22 (d, *J* = 7.5 Hz, 1H), 8.16 (d, *J* = 8.1 Hz, 2H), 8.07 (d, *J* = 8.5 Hz, 2H), 8.02 (d, *J* = 8.1 Hz, 1H), 8.00–7.94 (m, 4H), 7.87–7.79 (m, 5H), 7.59 (t, *J* = 7.8 Hz, 1H), 7.49 (t, *J* = 7.6 Hz, 1H), 3.06 (q, *J* = 7.2 Hz, 6H), 1.14 (t, *J* = 7.3 Hz, 9H); ^13^C-NMR (125 MHz, DMSO-*d*_6_): δ 165.1, 165.0, 147.1, 145.5, 142.0, 141.8, 140.7, 139.1, 135.8, 135.0, 134.5, 134.1, 133.3, 131.8, 128.8, 128.6, 128.2, 127.4, 127.3, 127.1, 125.9, 125.8, 125.3, 124.2, 124.1, 124.0, 123.0, 120.7, 45.8, 8.6; HRMS (ESI) [M − H]^−^ calcd. for C_36_H_24_N_3_O_7_S^−^, 642.1340; found, 642.1361.

*8-(4′-(4′-(Methoxycarbonyl)-[1,1′-biphenyl]-4-carboxamido)-[1,1′-biphenyl]-4-carboxamido)naphthalene-1-sulfonic acid* (**15**). The general procedure for the coupling reaction as described earlier was followed with 4′-(methoxycarbonyl)-[1,1′-biphenyl]-4-carboxylic acid (167 mg, 0.65 mmol) and 8-(4′-amino-[1,1′-biphenyl]-4-carboxamido)naphthalene-1-sulfonic acid **4** (260 mg, 0.50 mmol) to give the triethylamine salt of the title compound as a white solid (326 mg, 86%) (>99.3% pure by HPLC analysis (UV spectra at 254 nm)). ^1^H-NMR (500 MHz, DMSO-*d*_6_): *δ* 12.65 (s, 1H), 10.48 (s, 1H), 8.82 (br, 1H), 8.33 (d, *J* = 7.2 Hz, 1H), 8.28 (d, *J* = 8.0 Hz, 2H), 8.21 (d, *J* = 7.4 Hz, 1H), 8.14 (d, *J* = 8.1 Hz, 2H), 8.09 (d, *J* = 8.2 Hz, 2H), 8.02 (d, *J* = 8.0 Hz, 1H), 7.98 (d, *J* = 8.3 Hz, 2H), 7.97–7.91 (m, 4H), 7.89–7.78 (m, 5H), 7.59 (t, *J* = 7.8 Hz, 1H), 7.49 (t, *J* = 7.7 Hz, 1H), 3.90 (s, 3H), 3.06 (q, *J* = 7.2 Hz, 6H), 1.14 (t, *J* = 7.3 Hz, 9H); ^13^C-NMR (125 MHz, DMSO-*d*_6_): δ 166.0, 165.1, 143.6, 142.1, 141.8, 141.7, 139.2, 135.8, 134.5, 134.1, 133.3, 131.9, 129.9, 129.1, 128.8, 128.5, 127.4, 127.3, 127.1, 127.0, 125.9, 125.8, 125.3, 124.2, 124.0, 123.0, 120.7, 52.2, 45.8, 8.6; HRMS (ESI) [M + H]^+^ calcd. for C_38_H_29_N_2_O_7_S^+^, 657.1689; found, 657.1677.

*8-(2,2′-Dimethyl-4′-(4-nitrobenzamido)-[1,1′-biphenyl]-4-carboxamido)naphthalene-1-sulfonic acid (***16***)*. The general procedure for the coupling reaction as described earlier was followed with 4-nitrobenzoic acid **5** (251 mg, 1.5 mmol) and 8-(4′-amino-2,2′-dimethyl-[1,1′-biphenyl]-4-carboxamido)naphthalene-1-sulfonic acid (446 mg, 0.50 mmol) to give the triethylamine salt of the title compound as a yellow solid (608 mg, 87%) (>99.9% pure by HPLC analysis (UV spectra at 254 nm)). ^1^H-NMR (500 MHz, DMSO-*d*_6_): *δ* 12.61 (s, 1H), 10.59 (s, 1H), 8.80 (br, 1H), 8.39 (d, *J* = 8.7 Hz, 2H), 8.32 (d, *J* = 7.2 Hz, 1H), 8.22 (d, *J* = 8.9 Hz, 2H), 8.20 (d, *J* = 9.4 Hz, 1H), 8.14 (s, 1H),8.10 (d, *J* = 7.9 Hz, 1H), 8.01 (d, *J* = 8.2 Hz, 1H), 7.82 (d, *J* = 8.0 Hz, 1H), 7.79 (s, 1H), 7.71 (d, *J* = 8.2 Hz, 1H), 7.58 (t, *J* = 7.8 Hz, 1H), 7.48 (t, *J* = 7.6 Hz, 1H), 7.22 (d, *J* = 7.8 Hz, 1H), 7.15 (d, *J* = 8.2 Hz, 1H), 3.07 (q, *J* = 7.2 Hz, 6H), 2.13 (s, 3H), 2.08 (s, 3H), 1.15 (t, *J* = 7.3 Hz, 9H); ^13^C-NMR (125 MHz, DMSO-*d*_6_): δ 165.2, 163.9, 149.1, 143.4, 141.8, 140.7, 137.9, 136.5, 135.8, 135.4, 135.2, 134.5, 133.4, 131.8, 129.8, 129.2, 129.2, 129.1, 127.4, 125.9, 125.4, 125.3, 124.2, 123.9, 123.5, 123.0, 121.7, 117.9, 45.8, 19.8, 19.7, 8.6; HRMS (ESI) [M − H]^–^ calcd. for C_32_H_24_N_3_O_7_S^–^, 594.1340; found, 594.1336.

*8-(6-(4-Nitrobenzamido)-2-naphthamido)naphthalene-1-sulfonic acid* (**17**). The general procedure for the coupling reaction as described earlier was followed with 6-(4-nitrobenzamido)-2-naphthoic acid (168 mg, 0.5 mmol) and 8-aminonaphthalene-1-sulfonic acid **1** (223 mg, 0.5 mmol) to give the triethylamine salt of the title compound as a yellow solid (203 mg, 63%) (>99.9% pure by HPLC analysis (UV spectra at 254 nm)). ^1^H-NMR (500 MHz, DMSO-*d*_6_): *δ* 12.75 (s, 1H), 10.87 (s, 1H), 8.79 (br, 1H), 8.75 (s, 1H), 8.57 (s, 1H), 8.40 (d, *J* = 7.9 Hz, 2H), 8.32 (d, *J* = 7.2 Hz, 1H), 8.30–8.20 (m, 4H), 8.07 (d, *J* = 8.9 Hz, 1H), 8.02 (d, *J* = 8.1 Hz, 1H), 7.98 (d, *J* = 8.6 Hz, 1H), 7.93 (d, *J* = 8.9 Hz, 1H), 7.83 (d, *J* = 8.0 Hz, 1H), 7.59 (t, *J* = 7.7 Hz, 1H), 7.48 (t, *J* = 7.5 Hz, 1H), 3.06 (q, *J* = 7.1 Hz, 6H), 1.14 (t, *J* = 7.2 Hz, 9H); ^13^C-NMR (125 MHz, DMSO-*d*_6_): δ 165.4, 164.3, 149.2, 141.8, 140.5, 137.8, 135.8, 134.6, 133.4, 132.1, 131.9, 129.7, 129.3, 129.3, 128.3, 127.4, 127.2, 125.9, 125.6, 125.3, 124.1, 124.0, 123.5, 123.0, 121.1, 116.4, 45.8, 8.6; HRMS (ESI) [M − H]^−^ calcd. for C_28_H_18_N_3_O_7_S^−^, 540.0871; found, 540.0893.

*8-(4-(5-(4-Nitrobenzamido)pyridin-2-yl)benzamido)naphthalene-1-sulfonic acid* (**18**). The general procedure for the coupling reaction as described earlier was followed with 4-nitrobenzoic acid **5** (109 mg, 0.65 mmol) and 8-(4-(5-aminopyridin-2-yl)benzamido)naphthalene-1-sulfonic acid (260 mg, 0.50 mmol) to give the triethylamine salt of the title compound as a yellow solid (260 mg, 78%) (>99.6% pure by HPLC analysis (UV spectra at 254 nm)). ^1^H-NMR (500 MHz, DMSO-*d*_6_): *δ* 12.67 (s, 1H), 10.89 (s, 1H), 9.09 (s, 1H), 8.80 (br, 1H), 8.38 (d, *J* = 8.3 Hz, 2H), 8.36 (d, *J* = 8.1 Hz, 1H), 8.32 (d, *J* = 7.1 Hz, 1H), 8.28 (d, *J* = 8.0 Hz, 2H), 8.24 (d, *J* = 8.4 Hz, 2H), 8.21 (d, *J* = 8.1 Hz, 3H), 8.14 (d, *J* = 8.5 Hz, 1H), 8.01 (d, *J* = 8.0 Hz, 1H), 7.82 (d, *J* = 8.0 Hz, 1H), 7.58 (t, *J* = 7.8 Hz, 1H), 7.48 (t, *J* = 7.5 Hz, 1H), 3.07 (q, *J* = 6.4 Hz, 6H), 1.15 (t, *J* = 7.2 Hz, 9H); ^13^C-NMR (125 MHz, DMSO-*d*_6_): δ 165.1, 164.3, 150.6, 149.3, 141.8, 141.7, 140.5, 139.9, 135.8, 135.5, 134.9, 133.2, 131.9, 129.3, 128.5, 128.2, 127.5, 126.0, 125.7, 125.4, 124.3, 124.0, 123.6, 123.0, 120.5, 45.8, 8.6; HRMS (ESI) [M − H]^−^ calcd. for C_29_H_19_N_4_O_7_S^−^, 567.0980; found, 567.0998.

*8-(4-(5-(4-(Methoxycarbonyl)benzamido)pyridin-2-yl)benzamido)naphthalene-1-sulfonic acid* (**19**)*.* The general procedure for the coupling reaction as described earlier was followed with 4-(methoxycarbonyl)benzoic acid (117 mg, 0.65 mmol) and 8-(4-(5-aminopyridin-2-yl)benzamido)naphthalene-1-sulfonic acid (260 mg, 0.50 mmol) to give the triethylamine salt of the title compound as a white solid (227 mg, 66%) (>97.3% pure by HPLC analysis (UV spectra at 254 nm)). ^1^H-NMR (500 MHz, DMSO-*d*_6_): δ 12.67 (s, 1H), 10.79 (s, 1H), 9.10 (s, 1H), 8.80 (br, 1H), 8.37 (d, *J* = 8.6 Hz, 1H), 8.34–8.26 (m, 3H), 8.24–8.18 (m, 3H), 8.17–8.09 (m, 5H), 8.02 (d, *J* = 8.2 Hz, 1H), 7.82 (d, *J* = 8.0 Hz, 1H), 7.58 (t, *J* = 7.8 Hz, 1H), 7.48 (t, *J* = 7.6 Hz, 1H), 3.92 (s, 3H), 3.08 (dt, *J* = 12.4, 7.1 Hz, 6H), 1.15 (t, *J* = 7.3 Hz, 9H); ^13^C-NMR (125 MHz, DMSO-*d*_6_): δ 165.6, 165.1, 165.0, 150.4, 141.8, 141.7, 140.5, 138.4, 135.8, 135.5, 135.1, 133.3, 132.3, 131.8, 129.2, 128.5, 128.2, 127.4, 125.9, 125.7, 125.3, 124.2, 124.0, 123.0, 120.5, 52.4, 45.8, 8.6; HRMS (ESI) [M − H]^−^ calcd. for C_31_H_22_N_3_O_7_S^−^, 580.1184; found, 580.1201.

### 4.3. Binding Assays

Binding inhibition assays were performed in a 96-well cell-free format as described before [54,56,76]. Briefly, microtiter plates (Nunc F Maxisorp, 96-well; Thermo Fisher Scientific, Waltham, MA, USA) were coated overnight at 4 °C with 100 μL/well of Fc-conjugated receptors diluted in PBS pH 7.2. This was followed by blocking with 200 μL/well of blocking solution (PBS pH 7.2, 0.05% Tween-20, 1% BSA) for 1 h at RT. Then, plates were washed twice using washing solution (PBS pH 7.4, 0.05% Tween-20) and tapped dry before the addition of the appropriate FLAG tagged ligands along with different concentrations of tested dyes diluted in binding buffer (100 mM HEPES, 0.005% BSA pH 7.2) to give a total volume of 100 μL/well. After 1 h incubation, three washes were conducted, and anti-FLAG HRP conjugate was used to detect the bound FLAG-tagged ligand. Plates were washed four times before the addition of 120 μL/well of HRP substrate TMB (3,3′,5,5′-tetramethylbenzidine) and kept in the dark for up to 15 min. The reaction was stopped using 30 μL of 1M H_2_SO_4_, and the absorbance value was read at 450 nm. The concentrations of receptors used were 0.3 μg/mL for CD40, TNF-R1, and BAFFR; 0.6 μg/mL for OX40. The concentrations of the ligands were fixed at 0.02 μg/mL for CD154 and TNF-α, 0.06 μg/mL for OX40L, and 0.03 μg/mL for BAFF. These values were selected following preliminary testing to optimize response (i.e., to produce a high-enough signal at conditions close to half-maximal response, EC_50_). Stock solutions of compounds at 10 mM in DMSO were used; DMSO concentrations below 3% were found to not cause any effect on the readouts. To verify that inhibition is not due to colloidal aggregation, CD40 binding inhibition was also measured in the presence of the non-ionic detergent Triton-X 100 (0.01%), as recommended for the detection of such effects [60,61].

### 4.4. Protein Thermal Shift (Differential Scanning Fluorimetry)

This assay was used following standard protocols as described in the literature [63,64] to establish whether CD154 or CD40 is the binding partner of our compounds. Sypro orange (ThermoFisher; Waltham, MA, USA) was used as the fluorescence detection dye with an RT-PCR machine (StepOnePlus, Applied Biosystems (Foster City, CA, USA); detection on ROX channel, 575/602 nm) programmed to equilibrate samples at 25 °C for 90 s and then increase temperature to 99 °C by 0.4 °C every 24 s before taking a reading. Melting point of the protein is considered the lowest point of the first derivative plot, as calculated by the software included with the RT-PCR machine. Optimal concentrations were determined by performing a series of preliminary scans at various concentrations of protein, compound, and dye (CD154 0.05 mg/mL, CD40-Fc 0.1 mg/mL, Sypro orange 4×, 100 mM HEPES buffer, 10 μM of DRI-C21045 and 25 μM of **11**).

### 4.5. CD40 Sensor Cell Assay

CD40 expressing NF-κB sensor cells (HEK Blue, InvivoGen, San Diego, CA, USA) were used to assess the ability of the present compounds to block CD154-induced activation as described before [56,76,77]. Briefly, cells were maintained in DMEM at 80% confluence for each experiment. Cells were trypsinized and re-suspended in the same medium without FBS and seeded on 96-well microtiter plates at a density of 50,000 cells/well in the absence and presence of various concentrations of compounds diluted in the same media. For ligand mediated stimulation, a concentration of recombinant human CD154 (100 ng/mL) found to induce an optimal response was maintained in the wells for this purpose. After 24 h of incubation at 37 °C, 40 μL supernatant of each well were taken and added to another 96-well microtiter plate containing 160 μL/well of QUANTI-Blue (InvivoGen). The level of SEAP was determined after 1 h of incubation at 37 °C by reading at 650 nm using a spectrophotometer. A monoclonal anti-human CD154 antibody (clone 40804, R&D Systems, Minneapolis, MN, USA) was used as a positive control at 240 nM concentration.

### 4.6. Cytotoxicity Assay

For the MTS assay, HEK Blue cells were cultured and prepared in the same manner as in the CD40 sensor assay. Then cells were added to a 96-well microtiter plate at a density of 50,000 cells/well in the absence or presence of various concentrations of compounds diluted in the same media, without the addition of CD154. The plate was incubated at 37 °C for 24 h. 20 µL per well of MTS, 3-(4,5-dimethylthiazol-2-yl)-5-(3-carboxymethoxyphenyl)-2-(4-sulfophenyl)-2H-tetrazolium, (Promega, Madison, WI, USA) was added to the culture after treatments, and cells were incubated at 37 °C for 4 h. Formazan levels were measured using a plate reader at 490 nm.

### 4.7. Animal Care and Treatment

All mice used for these studies (C57BL/6, BALB/c, and DBA-2) were obtained from Jackson Laboratories (Bar Harbor, ME, USA). All animal studies were reviewed and approved by the University of Miami Institutional Animal Care and Use Committee. Procedures were conducted according to the guidelines of the Committee on Care and Use of Laboratory Animals, Institute of Laboratory Animal Resources (National Research Council, Washington, DC, USA). Animals were housed in microisolated cages in Virus Antibody Free rooms with free access to autoclaved food and water at the Department of Veterinary Resources of the University of Miami.

### 4.8. Draining Lymph Node

The ability to inhibit alloantigen-induced T cell response in a draining lymph node was tested as described before [56]. In brief, BALB/c mice (10–12 weeks old, female) received a footpad injection of 1 × 10^7^ splenocytes isolated from DBA-2 mice (10–12 weeks old, male). CD154 antibody (MR-1; 20 mg/kg, day −1 and 0) or test compounds (20–60 mg/kg; b.i.d. from day −1 to 3, in 20% HPβCD) were administered s.c., and the draining popliteal lymph nodes (DLNs) were collected 3 days after the alloantigen challenge. DLN cells were counted and data are shown as mean ± SD (*n* = 3–4 per group).

### 4.9. Statistics and Data Fitting

All binding inhibition and cell assays were tested in duplicate or triplicate per plates, and assays were performed as at least three independent experiments. As before [56,62,76], binding and cytotoxicity data were converted to percent inhibition and fitted with standard log inhibitor vs. normalized response models [78] using nonlinear regression in GraphPad Prism (GraphPad, La Jolla, CA, USA) to establish half-maximal (median) inhibitory concentration (IC_50_) or median lethal concentration (LC_50_) values. Cell assay data were fitted with a similar model that also allowed for a variable slope using the log inhibitor vs. normalized response (variable slope) model from Prism with a Hill slope (*n*) shared across all compounds. Cell assays and draining lymph node data were analyzed by one-way repeated-measures analysis of variance (ANOVA) followed by Dunnett’s multiple comparison test as a *post hoc* test for individual differences using GraphPad Prism and a significance level of *p* < 0.05 for all comparisons.

## 5. Conclusions

In conclusion, we have identified a set of new drug-like small-molecule inhibitors of the CD40–CD154 interaction with activity confirmed in cell-based assays and a mouse draining lymph node experiment. Our results expand the chemical space of identified small-molecule CD40–C154 costimulatory inhibitors and provide further evidence that this costimulatory PPI is susceptible to small-molecule inhibition. These lead structures have the potential to be developed as orally bioavailable immunomodulatory therapeutics that are safer and less likely to face immunogenicity problems than protein-based biologics.

## Figures and Tables

**Table 1:** Summary of data for compounds of the present study.

No.	Name	Formula	R_1_^a^	R_2_^a^	CD40 IC_50_ [μM]	OX40 IC_50_ [μM]	BAFF IC_50_ [μM]	TNF IC_50_ [μM]	NF-κB IC_50_ [μM]
**6**	DRI-C21041	C_30_H_21_N_3_O_7_S	1-SO_3_H	4′-NO_2_	0.087	317.9	0.328	>1000	19.9
**7**	DRI-C24041	C_30_H_21_N_3_O_7_S	4-SO_3_H	4′-NO_2_	0.299	9.34	2.007	554.6	
**8**	DRI-21047a	C_31_H_21_F_3_N_2_O_5_S	1-SO_3_H	4′-CF_3_	57.85	10.07	42.18	61.07	
**9**	DRI-C24541	C_32_H_23_N_3_O_6_	4-CO_2_Me	4′-NO_2_	55.99	>1000	685.6	795.8	
**10**	DRI-C2105041	C_30_H_21_N_3_O_10_S_2_	1,5-(SO_3_H)_2_	4′-NO_2_	68.31	135.0	439.7	688.5	
**11**	DRI-C2105045	C_32_H_24_N_2_O_10_S_2_	1,5-(SO_3_H)_2_	4′-CO_2_Me	60.07	120.4	540.1	456.1	
**12**	DRI-C2104121	C_30_H_20_N_4_O_9_S	1-SO_3_H	2′,4′-(NO_2_)_2_	50.50	10.31	38.14	61.68	
**13**	DRI-C2104531	C_32_H_23_N_3_O_9_S	1-SO_3_H	3′-NO_2_-4′-CO_2_Me	0.390	86.19	5.27	674.4	
**14**	DRI-C21091	C_36_H_25_N_3_O_7_S	1-SO_3_H	4′-(4-NO_2_)C_6_H_4_	0.020	6.88	0.784	70.35	11.1
**15**	DRI-C21095	C_38_H_28_N_2_O_7_S	1-SO_3_H	4′-(4-CO_2_Me)C_6_H_4_	0.019	1.013	1.517	26.55	6.0
**16**	DRI-C31041	C_32_H_25_N_3_O_7_S	1-SO_3_H	4′-NO_2_	44.80	7.28	26.73	61.48	
**17**	DRI-C41041	C_28_H_19_N_3_O_7_S	1-SO_3_H	4′-NO_2_	57.45	36.63	81.38	118.5	
**18**	DRI-C61041	C_29_H_20_N_4_O_7_S	1-SO_3_H	4′-NO_2_	1.34	5.48	8.07	46.94	8.7
**19**	DRI-C61045	C_31_H_23_N_3_O_7_S	1-SO_3_H	4′-CO_2_Me	2.41	6.97	13.76	182.0	

^a^ For numbering, see structure **6** in Scheme 1.

## Abbreviations

The following abbreviations are used in this manuscript:

CI	confidence interval
PPI	protein-protein interaction
T1D	type 1 diabetes
TNF	tumor necrosis factor
TNFSF	TNF superfamily
